# Supplementary material for: A longitudinal study of the diabetic skin and wound microbiome
Source: PeerJ. 2017 Jul 20;5:e3543. doi: 10.7717/peerj.3543 (PMC5522608; doi:10.7717/peerj.3543)
Supplement: Table S1 — Sample types are indicated in the second column: skin swab from an area adjacent to the foot wound (SA), skin swab from the contralateral foot (SC), wound swab (WS) and debrided wound tissue (WD). A tick (x) indicates that a sample was collected, a cross (x) indicates that no samples for that time point were collected because the patient could not make their scheduled appointment, and NC indicates that a sample was not collected because podiatry staff deemed there was not sufficient tissue available for a debridement sample. In the case that a wound healed (indicated by WH) no further samples were collected for that patient. In the case of P4, the contralateral foot had previously been amputated, and the foot containing the wound was amputated after time point 2. Samples for which a 16S rRNA gene PCR product was not obtained are indicated with (x). [file peerj-05-3543-s001.docx]

| Time point | Subject  Sample | P1 | P2 | P3 | P4 | P5 | P6 | P7 | P8 | P9 | P10 |
| --- | --- | --- | --- | --- | --- | --- | --- | --- | --- | --- | --- |
| 0 | SA | ✓ | ✓ | ✓ | ✓ | ✓ | ✓ | ✓ | ✓ | ✓ | ✓ |
|  | SC | ✓ | ✓ | ✓ | NC | ✓ | ✓ | ✓ | ✓ | ✓ | ✓ |
|  | WS | ✓ | ✓ | ✓ | ✓(x) | ✓ | ✓ | ✓ | ✓ | ✓ | ✓ |
|  | WD | ✓ | ✓ | ✓ | ✓(x) | ✓ | NC | ✓ | ✓ | ✓ | ✓ |
| 1 | SA | ✓ | ✕ | ✓ | ✓ | ✓ | ✓ | ✓ | ✓ | ✓ | ✓ |
|  | SC | ✓ | ✕ | ✓ | NC | ✓ | ✓ | ✓ | ✓ | ✓ | ✓ |
|  | WS | ✓ | ✕ | ✓ | ✓(x) | ✓ | ✓ | ✓ | ✓ | ✓ | ✓ |
|  | WD | ✓ | ✕ | ✓ | ✓(x) | ✓ | ✓ | ✓ | ✓ | ✓ | ✓ |
| 2 | SA | ✓ | ✕ | ✓ | ✓ | ✓ | ✓ | ✓ | ✓ | ✓ | ✓ |
|  | SC | ✓ | ✕ | ✓ | NC | ✓ | ✓ | ✓ | ✓ | ✓ | ✓ |
|  | WS | ✓ | ✕ | WH | ✓(x) | ✓ | ✓ | ✓ | ✓ | ✓ | ✓ |
|  | WD | ✓ | ✕ | WH | ✓(x) | ✓ | ✓ | ✓ | ✓ | ✓ | ✓ |
| 3 | SA | ✓ | ✓ |  |  | ✓ | ✓ | ✓ | ✓ | ✓ | ✓ |
|  | SC | ✓ | ✓ |  |  | ✓ | ✓ | ✓ | ✓ | ✓ | ✓ |
|  | WS | ✓ | ✓ |  |  | ✓ | ✓ | ✓ | ✓ | ✓(x) | ✓ |
|  | WD | ✓ | NC |  |  | ✓ | NC | NC | ✓ | NC | ✓ |
| 4 | SA | ✓ | ✓ |  |  | ✓ | ✓ | ✓ | ✕ | ✓ | ✓ |
|  | SC | ✓ | ✓ |  |  | ✓ | ✓ | ✓ | ✕ | ✓ | ✓ |
|  | WS | ✓ | ✓ |  |  | ✓ | ✓ | WH | ✕ | ✓ | ✓ |
|  | WD | ✓ | NC |  |  | NC | ✓ | WH | ✕ | NC | ✓ |
| 5 | SA | ✓ | ✓ |  |  | ✓ | ✓ |  | ✓ | ✓ | ✕ |
|  | SC | ✓ | ✓ |  |  | ✓ | ✓ |  | ✓ | ✓ | ✕ |
|  | WS | ✓ | ✓ |  |  | ✓ | ✓(x) |  | ✓ | ✓ | ✕ |
|  | WD | ✓ | NC |  |  | ✓ | NC |  | ✓ | ✓ | ✕ |

**Table S1: Summary of samples collected from each diabetic patient enrolled in the study.** Sample types are indicated in the second column: skin swab from an area adjacent to the foot wound (SA), skin swab from the contralateral foot (SC), wound swab (WS) and debrided wound tissue (WD). A tick (✓) indicates that a sample was collected, a cross (✕) indicates that no samples for that time point were collected because the patient could not make their scheduled appointment, and NC indicates that a sample was not collected because podiatry staff deemed there was not sufficient tissue available for a debridement sample. In the case that a wound healed (indicated by WH) no further samples were collected for that patient. In the case of P4, the contralateral foot had previously been amputated, and the foot containing the wound was amputated after time point 2. Samples for which a 16S rRNA gene PCR product was not obtained are indicated with (x).
