# Supplementary material for: A longitudinal study of the diabetic skin and wound microbiome
Source: PeerJ. 2017 Jul 20;5:e3543. doi: 10.7717/peerj.3543 (PMC5522608; doi:10.7717/peerj.3543)
Supplement: Table S2 — Sample types are indicated in the second column: skin swab from the base of the left foot (L), or right foot (F). A tick (x) indicates that a sample was collected, a cross (x) indicates that no samples for that time point were collected because the subject was not available. Samples for which a 16S rRNA gene PCR product was not obtained are indicated with (x). Control subject 8 (CP8) was removed from the study as not enough samples were collected. [file peerj-05-3543-s002.docx]

| Time point | Subject  Sample | CP1 | CP2 | CP3 | CP4 | CP5 | CP6 | CP7 | CP8 | CP9 |
| --- | --- | --- | --- | --- | --- | --- | --- | --- | --- | --- |
| 0 | L | ✓ | ✓ | ✓(x) | ✓ | ✓ | ✓(x) | ✓ | ✕ | ✓ |
|  | R | ✓ | ✓ | ✓(x) | ✓ | ✓ | ✓(x) | ✓(x) | ✕ | ✓ |
| 1 | L | ✓ | ✓ | ✓ | ✓(x) | ✓ | ✓ | ✓ | ✓ | ✓ |
|  | R | ✓ | ✓ | ✓ | ✓(x) | ✓ | ✓ | ✓ | ✓ | ✓ |
| 2 | L | ✓ | ✓ | ✓ | ✓ | ✓ | ✓ | ✓ | ✓ | ✓ |
|  | R | ✓ | ✓ | ✓ | ✓ | ✓ | ✓ | ✓ | ✓ | ✓ |
| 3 | L | ✓ | ✓ | ✓ | ✓ | ✓ | ✓ | ✓ | ✕ | ✓ |
|  | R | ✓ | ✓ | ✓ | ✓ | ✓ | ✓ | ✓ | ✕ | ✓ |
| 4 | L | ✓ | ✓ | ✓ | ✓ | ✓ | ✓ | ✓ | ✕ | ✓ |
|  | R | ✓ | ✓ | ✓ | ✓ | ✓ | ✓ | ✓(x) | ✕ | ✓ |
| 5 | L | ✓ | ✓ | ✕ | ✓ | ✓ | ✓ | ✓ | ✕ | ✓ |
|  | R | ✓ | ✓ | ✕ | ✓ | ✓ | ✓ | ✓ | ✕ | ✓ |

**Table S2: Summary of samples collected from each control subject enrolled in the study.** Sample types are indicated in the second column: skin swab from the base of the left foot (L), or right foot (F). A tick (✓) indicates that a sample was collected, a cross (✕) indicates that no samples for that time point were collected because the subject was not available. Samples for which a 16S rRNA gene PCR product was not obtained are indicated with (x). Control subject 8 (CP8) was removed from the studies as not enough samples were collected.
