# Supplementary material for: A longitudinal study of the diabetic skin and wound microbiome
Source: PeerJ. 2017 Jul 20;5:e3543. doi: 10.7717/peerj.3543 (PMC5522608; doi:10.7717/peerj.3543)
Supplement: Table S3 — Wound size is shown as height × width × depth in mm. All wounds were routinely irrigated with Prontosan solution, followed by dressing with either Allevyn foam for moist wound healing, Zetuvit dressing to remove excess wound exudate, Inadine antimicrobial dressing (10% povidone-iodine), or Acticoat flex (silver coated antimicrobial dressing). Decisions on wound dressing were made by the treating podiatrist or wound care nurse. [file peerj-05-3543-s003.docx]

|  |  | **Time** | | | | | |  |
| --- | --- | --- | --- | --- | --- | --- | --- | --- |
| **Patient** |  | **0** | **1** | **2** | **3** | **4** | **5** | **Location** |
| 1 | treatment | Prontasan, Allevyn | Prontasan, Allevyn | Prontasan, Allevyn | Prontasan, Allevyn | Prontasan, Allevyn | Prontasan, Allevyn | Left 5th metatarsophalangeal joint |
|  | wound size (mm) | 10 x 11 x 1 | 8 x 8 x 2 | 8 x 3 x 2 | 8 x 3 x 2 | 7 x 2 x 1 | 6 x 3 x 1 |  |
| 2 | treatment | Prontasan,  Inadine | x | x | Prontasan,  Inadine | Prontasan,  Inadine | Prontasan,  Inadine | Left 5th distal phalanx |
|  | wound size (mm) | 4 x 1 x 1 | x | x | 2 x 1 x 1 | 2 x 1 x 1 | 3 x 2 x 2 |  |
| 5 | treatment | Prontasan, Allevyn | Prontasan, Allevyn | Prontasan, Allevyn | Prontasan, Allevyn | Prontasan, Allevyn | Prontasan, Allevyn | Right 2nd distal phalanx |
|  | wound size (mm) | 3 x 2 x 1 | 3 x 2 x 1 | 8 x 6 x 1 | 2 x 2 x 1 | 5 x 3 x 1 | 5 x 5 x 1 |  |
| 6 | treatment | Prontasan, Allevyn | Prontasan, Allevyn | Prontasan, Allevyn | Prontasan, Allevyn |  | x | Right 1st distal phalanx |
|  | wound size (mm) | 4 x 1 x 3.5 | 5 x 1 x 2 | 4 x 1 x 2 | 3 x 1 x 2 | (healed) | (healed) |  |
| 7 | treatment | Prontosan, Inadine | Prontosan, Allevyn | Prontosan, Allevyn | x | x | x | Right 2nd metatarsophalangeal joint |
|  | wound size (mm) | 12 x 6 x 2 | 11 x 6 x 2 | 6 x 6 x 3 | (healed) | (healed) | (healed) |  |
| 8 | treatment | Prontosan, Inadine | Prontosan, Inadine | Prontosan, Inadine | Prontosan, Inadine | x | Prontosan, Inadine | Left 3rd metatarsophalangeal joint |
|  | wound size (mm) | 4 x 4 x 4 | 8 x 6 x 4 | 7 x 5 x 1 | 8 x 5 x 1 | x | 8 x 5 x 1 |  |
| 9 | treatment | Prontosan, Acticoat flex | x | Prontosan, Acticoat flex | Prontosan, Acticoat flex | Prontosan, Acticoat flex | Prontosan, Acticoat flex | Left 2nd metatarsal head |
|  | wound size (mm) | 20 x 10 x 1 | x | 22 x 10 x 1 | 22 x 10 x 1 | 22 x 10 x 1 | 19 x 9 x 1 |  |
| 10 | treatment | Prontosan, Allevyn | Prontosan, Allevyn | Prontosan, Zetuvit | Prontosan, Zetuvit | Prontosan, Acticoat flex | x | Right 2nd metatarsal phalangeal joint |
|  | wound size (mm) | 7 x 5 x 12 | 8 x 4 x 12 | 4 x 3 x 12 | 6 x 3 x 12 | 4 x 4 x 12 | x |  |

**Table S3: Patient wound location, size and treatment over time.** Wound size is shown as height x width x depth in mm. All wounds were routinely irrigated with Prontosan solution, followed by dressing with either Allevyn foam for moist wound healing, Zetuvit dressing to remove excess wound exudate, Inadine antimicrobial dressing (10% povidone-iodine), or Acticoat flex (silver coated antimicrobial dressing). Decisions on wound dressing were made by the treating podiatrist or wound care nurse.
