# Supplementary material for: A longitudinal study of the diabetic skin and wound microbiome
Source: PeerJ. 2017 Jul 20;5:e3543. doi: 10.7717/peerj.3543 (PMC5522608; doi:10.7717/peerj.3543)
Supplement: Table S4 — Coverage before and after quality filtering for each sample is indicated, along with the sequencing run that each sample was sequenced in. [file peerj-05-3543-s004.docx]

| **Sample** | **Sequence coverage prior to quality filtering** | **Sequence coverage after quality filtering** | **Sequencing run** |
| --- | --- | --- | --- |
| CP1.0_L | 55444 | 45384 | 1 |
| CP1.0_R | 84263 | 69590 | 1 |
| CP1.1_L | 89043 | 71323 | 1 |
| CP1.1_R | 47765 | 38249 | 1 |
| CP1.2_L | 95861 | 76438 | 1 |
| CP1.2_R | 67736 | 54679 | 1 |
| CP1.3_L | 64711 | 55058 | 1 |
| CP1.3_R | 30945 | 23779 | 1 |
| CP1.4_L | 110418 | 90891 | 1 |
| CP1.4_R | 103019 | 88643 | 1 |
| CP1.5_L | 73570 | 57642 | 1 |
| CP1.5_R | 73720 | 58868 | 1 |
| CP2.0_L | 62935 | 46830 | 1 |
| CP2.0_R | 65327 | 46722 | 1 |
| CP2.1_L | 74665 | 62021 | 1 |
| CP2.1_R | 89511 | 70903 | 1 |
| CP2.2_L | 65302 | 55118 | 1 |
| CP2.2_R | 67888 | 54550 | 1 |
| CP2.3_L | 82749 | 29519 | 1 |
| CP2.3_R | 55029 | 14781 | 1 |
| CP2.4_L | 82207 | 69645 | 1 |
| CP2.4_R | 50338 | 43739 | 1 |
| CP2.5_L | 74249 | 62887 | 1 |
| CP2.5_R | 63885 | 50520 | 1 |
| CP3.1_L | 110423 | 91383 | 2 |
| CP3.1_R | 155581 | 135902 | 2 |
| CP3.2_L | 132733 | 116941 | 2 |
| CP3.2_R | 109240 | 89396 | 2 |
| CP3.3_L | 100605 | 90732 | 2 |
| CP3.3_R | 285756 | 258143 | 2 |
| CP3.4_L | 89837 | 82518 | 2 |
| CP3.4_R | 87600 | 80349 | 2 |
| CP4.0_L | 63696 | 51670 | 2 |
| CP4.0_R | 128448 | 103693 | 2 |
| CP4.2_L | 89546 | 73676 | 2 |
| CP4.2_R | 82529 | 65633 | 2 |
| CP4.3_L | 106719 | 86113 | 2 |
| CP4.3_R | 77997 | 65116 | 2 |
| CP4.4_L | 84727 | 72079 | 2 |
| CP4.4_R | 95062 | 83660 | 2 |
| CP4.5_L | 99216 | 89261 | 2 |
| CP4.5_R | 90581 | 80390 | 2 |
| CP5.0_L | 87801 | 71491 | 2 |
| CP5.0_R | 79818 | 65495 | 2 |
| CP5.1_L | 145413 | 125616 | 2 |
| CP5.1_R | 87820 | 76025 | 2 |
| CP5.2_L | 60769 | 49993 | 2 |
| CP5.2_R | 65475 | 54925 | 2 |
| CP5.3_L | 59724 | 53992 | 2 |
| CP5.3_R | 132611 | 116434 | 2 |
| CP5.4_L | 84174 | 72442 | 2 |
| CP5.4_R | 77937 | 68107 | 2 |
| CP5.5_L | 49990 | 49177 | 2 |
| CP5.5_R | 49990 | 42688 | 2 |
| CP6.1_L | 84679 | 66617 | 1 |
| CP6.1_R | 58397 | 49264 | 1 |
| CP6.2_L | 53550 | 43770 | 1 |
| CP6.2_R | 74145 | 58107 | 1 |
| CP6.3_L | 63020 | 49511 | 1 |
| CP6.3_R | 75568 | 57228 | 1 |
| CP6.4_L | 62865 | 44929 | 1 |
| CP6.4_R | 108011 | 89549 | 1 |
| CP6.5_L | 79727 | 52508 | 1 |
| CP6.5_R | 38756 | 26811 | 1 |
| CP7.0_L | 103695 | 88457 | 1 |
| CP7.1_L | 58769 | 49717 | 1 |
| CP7.1_R | 96507 | 84048 | 1 |
| CP7.2_L | 79398 | 65197 | 1 |
| CP7.2_R | 10637 | 8839 | 1 |
| CP7.3_L | 141834 | 118152 | 1 |
| CP7.3_R | 69855 | 59522 | 1 |
| CP7.4_L | 94425 | 80685 | 1 |
| CP7.5_L | 82940 | 71078 | 1 |
| CP7.5_R | 84512 | 74187 | 1 |
| CP9.0_L | 81789 | 69144 | 2 |
| CP9.0_R | 60361 | 52130 | 2 |
| CP9.1_L | 66833 | 59106 | 2 |
| CP9.1_R | 72924 | 60935 | 2 |
| CP9.2_L | 86340 | 75067 | 2 |
| CP9.2_R | 56695 | 50358 | 2 |
| CP9.3_L | 67639 | 58255 | 2 |
| CP9.3_R | 67552 | 59024 | 2 |
| CP9.4_L | 51560 | 43665 | 2 |
| CP9.4_R | 64568 | 55687 | 2 |
| CP9.5_L | 66161 | 55019 | 2 |
| CP9.5_R | 65068 | 52147 | 2 |
| P1.0_SA | 67537 | 60996 | 2 |
| P1.0_SC | 72453 | 63297 | 2 |
| P1.0_WD | 73177 | 63589 | 2 |
| P1.0_WS | 82017 | 72508 | 2 |
| P1.1_SA | 76323 | 67917 | 2 |
| P1.1_SC | 80383 | 68320 | 2 |
| P1.1_WD | 48223 | 42818 | 2 |
| P1.1_WS | 68975 | 62394 | 2 |
| P1.2_SA | 57928 | 51612 | 2 |
| P1.2_SC | 54216 | 46360 | 2 |
| P1.2_WD | 48165 | 40659 | 2 |
| P1.2_WS | 64606 | 55488 | 2 |
| P1.3_SA | 64012 | 58431 | 2 |
| P1.3_SC | 66002 | 57920 | 2 |
| P1.3_WD | 66100 | 51494 | 2 |
| P1.3_WS | 66604 | 54064 | 2 |
| P1.4_SA | 69741 | 62453 | 2 |
| P1.4_SC | 74771 | 66092 | 2 |
| P1.4_WD | 43892 | 35920 | 2 |
| P1.4_WS | 57474 | 47502 | 2 |
| P1.5_SA | 62492 | 55364 | 2 |
| P1.5_SC | 77242 | 68390 | 2 |
| P1.5_WD | 73603 | 61759 | 2 |
| P1.5_WS | 57480 | 48580 | 2 |
| P10.0_SA | 74355 | 64454 | 1 |
| P10.0_SC | 48051 | 41028 | 1 |
| P10.0_WD | 61469 | 72893 | 1 |
| P10.0_WS | 43164 | 35038 | 1 |
| P10.1_SA | 104704 | 90519 | 1 |
| P10.1_SC | 104817 | 88625 | 1 |
| P10.1_WD | 61469 | 49400 | 1 |
| P10.1_WS | 65707 | 53022 | 1 |
| P10.2_SA | 76786 | 66638 | 1 |
| P10.2_SC | 95289 | 80571 | 1 |
| P10.2_WD | 49507 | 37844 | 1 |
| P10.2_WS | 73836 | 58633 | 1 |
| P10.3_SA | 47634 | 39794 | 1 |
| P10.3_SC | 61602 | 52724 | 1 |
| P10.3_WD | 82379 | 65626 | 1 |
| P10.3_WS | 84424 | 67461 | 1 |
| P10.4_SA | 56053 | 47226 | 1 |
| P10.4_SC | 121554 | 100840 | 1 |
| P10.4_WD | 70303 | 56998 | 1 |
| P10.4_WS | 59344 | 46974 | 1 |
| P2.0_SA | 86382 | 76615 | 2 |
| P2.0_SC | 66135 | 56605 | 2 |
| P2.0_WD | 32645 | 27617 | 2 |
| P2.0_WS | 38868 | 34083 | 2 |
| P2.3_SA | 62455 | 56007 | 2 |
| P2.3_SC | 1683 | 1295 | 2 |
| P2.3_WS | 42254 | 33690 | 2 |
| P2.4_SA | 63727 | 55628 | 2 |
| P2.4_SC | 56861 | 46361 | 2 |
| P2.4_WS | 27692 | 22456 | 2 |
| P2.5_SA | 66292 | 58974 | 2 |
| P2.5_SC | 74018 | 61479 | 2 |
| P2.5_WS | 43613 | 38830 | 2 |
| P5.0_SA | 123979 | 107155 | 2 |
| P5.0_SC | 70325 | 56614 | 2 |
| P5.0_WD | 46225 | 38816 | 2 |
| P5.0_WS | 60372 | 52298 | 2 |
| P5.1_SA | 89447 | 80898 | 2 |
| P5.1_SC | 7865 | 6769 | 2 |
| P5.1_WD | 59962 | 52485 | 2 |
| P5.1_WS | 67488 | 56112 | 2 |
| P5.2_SA | 112463 | 98592 | 2 |
| P5.2_SC | 189865 | 172134 | 2 |
| P5.2_WD | 99624 | 89902 | 2 |
| P5.2_WS | 85698 | 77723 | 2 |
| P5.3_SA | 78209 | 67582 | 2 |
| P5.3_SC | 86947 | 77965 | 2 |
| P5.3_WD | 69686 | 60154 | 2 |
| P5.3_WS | 81724 | 72034 | 2 |
| P5.4_SA | 60855 | 53451 | 2 |
| P5.4_SC | 58193 | 47617 | 2 |
| P5.4_WS | 62080 | 55167 | 2 |
| P5.5_SA | 81949 | 72723 | 2 |
| P5.5_SC | 61245 | 53537 | 2 |
| P5.5_WD | 78103 | 65311 | 2 |
| P5.5_WS | 69365 | 54436 | 2 |
| P6.0_SA | 80777 | 66017 | 2 |
| P6.0_SC | 68520 | 58441 | 2 |
| P6.0_WS | 134413 | 117229 | 2 |
| P6.1_SA | 60233 | 50832 | 2 |
| P6.1_SC | 70791 | 58745 | 2 |
| P6.1_WD | 125202 | 103877 | 2 |
| P6.1_WS | 99210 | 80362 | 2 |
| P6.2_SA | 73753 | 62404 | 2 |
| P6.2_SC | 70484 | 50820 | 2 |
| P6.2_WD | 92729 | 78927 | 2 |
| P6.2_WS | 62052 | 51925 | 2 |
| P6.3_SA | 92038 | 80474 | 2 |
| P6.3_SC | 72012 | 62048 | 2 |
| P6.3_WS | 92084 | 78066 | 2 |
| P6.4_SA | 80471 | 68888 | 2 |
| P6.4_SC | 69616 | 59650 | 2 |
| P6.4_WD | 5677 | 4740 | 2 |
| P6.4_WS | 64604 | 56922 | 2 |
| P6.5_SA | 49657 | 43798 | 2 |
| P6.5_SC | 61936 | 51811 | 2 |
| P7.0_SA | 75328 | 64727 | 1 |
| P7.0_SC | 72281 | 58918 | 1 |
| P7.0_WD | 104285 | 84481 | 1 |
| P7.0_WS | 76386 | 62275 | 1 |
| P7.1_SA | 79225 | 68006 | 1 |
| P7.1_SC | 95741 | 75817 | 1 |
| P7.1_WD | 91012 | 78048 | 1 |
| P7.1_WS | 91651 | 74530 | 1 |
| P7.2_SA | 68857 | 59350 | 1 |
| P7.2_SC | 82420 | 66138 | 1 |
| P7.2_WD | 86018 | 74119 | 1 |
| P7.2_WS | 114869 | 97737 | 1 |
| P7.3_SA | 96275 | 82562 | 1 |
| P7.3_SC | 83660 | 67079 | 1 |
| P7.3_WS | 87395 | 75120 | 1 |
| P7.4_SA | 213653 | 178543 | 1 |
| P7.4_SC | 57379 | 48073 | 1 |
| P8.0_SA | 68574 | 57752 | 1 |
| P8.0_SC | 297817 | 254252 | 1 |
| P8.0_WD | 33065 | 27566 | 1 |
| P8.0_WS | 60546 | 47594 | 1 |
| P8.1_SA | 71865 | 60652 | 1 |
| P8.1_SC | 92130 | 74949 | 1 |
| P8.1_WD | 69051 | 59024 | 1 |
| P8.1_WS | 81260 | 63922 | 1 |
| P8.2_SA | 73594 | 63966 | 1 |
| P8.2_SC | 70653 | 61478 | 1 |
| P8.2_WD | 88455 | 69068 | 1 |
| P8.2_WS | 88678 | 69694 | 1 |
| P8.3_SA | 94335 | 78626 | 1 |
| P8.3_SC | 64088 | 53094 | 1 |
| P8.3_WD | 88372 | 71219 | 1 |
| P8.3_WS | 58458 | 45121 | 1 |
| P8.5_SA | 90561 | 74218 | 1 |
| P8.5_SC | 104034 | 82470 | 1 |
| P8.5_WD | 102774 | 80580 | 1 |
| P8.5_WS | 71173 | 54080 | 1 |
| P9.0_SA | 73236 | 62693 | 1 |
| P9.0_SC | 75985 | 63167 | 1 |
| P9.0_WD | 46079 | 36877 | 1 |
| P9.0_WS | 90613 | 73491 | 1 |
| P9.1_SA | 61651 | 52926 | 1 |
| P9.1_SC | 84035 | 70244 | 1 |
| P9.1_WD | 109176 | 95418 | 1 |
| P9.1_WS | 93959 | 75940 | 1 |
| P9.2_SA | 94330 | 82384 | 1 |
| P9.2_SC | 85045 | 74259 | 1 |
| P9.2_WD | 62755 | 50228 | 1 |
| P9.2_WS | 87888 | 68167 | 1 |
| P9.3_SA | 134321 | 116201 | 1 |
| P9.3_SC | 96602 | 82003 | 1 |
| P9.4_SA | 73746 | 62928 | 1 |
| P9.4_SC | 64982 | 55722 | 1 |
| P9.4_WS | 67440 | 58836 | 1 |
| P9.5_SA | 78138 | 67014 | 1 |
| P9.5_SC | 39374 | 34127 | 1 |
| P9.5_WD | 56229 | 45463 | 1 |
| P9.5_WS | 50125 | 37283 | 1 |
| Blank_swab_1 | 1508 | 1220 | 1 |
| Blank_swab_2 | 1855 | 1498 | 2 |
| Positive_E.coli | 71016 | 480739 | 1 |
| Positive_E.coli_2 | 566184 | 57240 | 2 |
| No_DNA | 4377 | 3622 | 1 |
| No_DNA_2 | 27840 | 24347 | 2 |
