# Supplementary figures and images for: A longitudinal study of the diabetic skin and wound microbiome
Source: PeerJ. 2017 Jul 20;5:e3543. doi: 10.7717/peerj.3543 (PMC5522608; doi:10.7717/peerj.3543)

# Unifrac distances between adjacent or contralateral diabetic skin and wounds

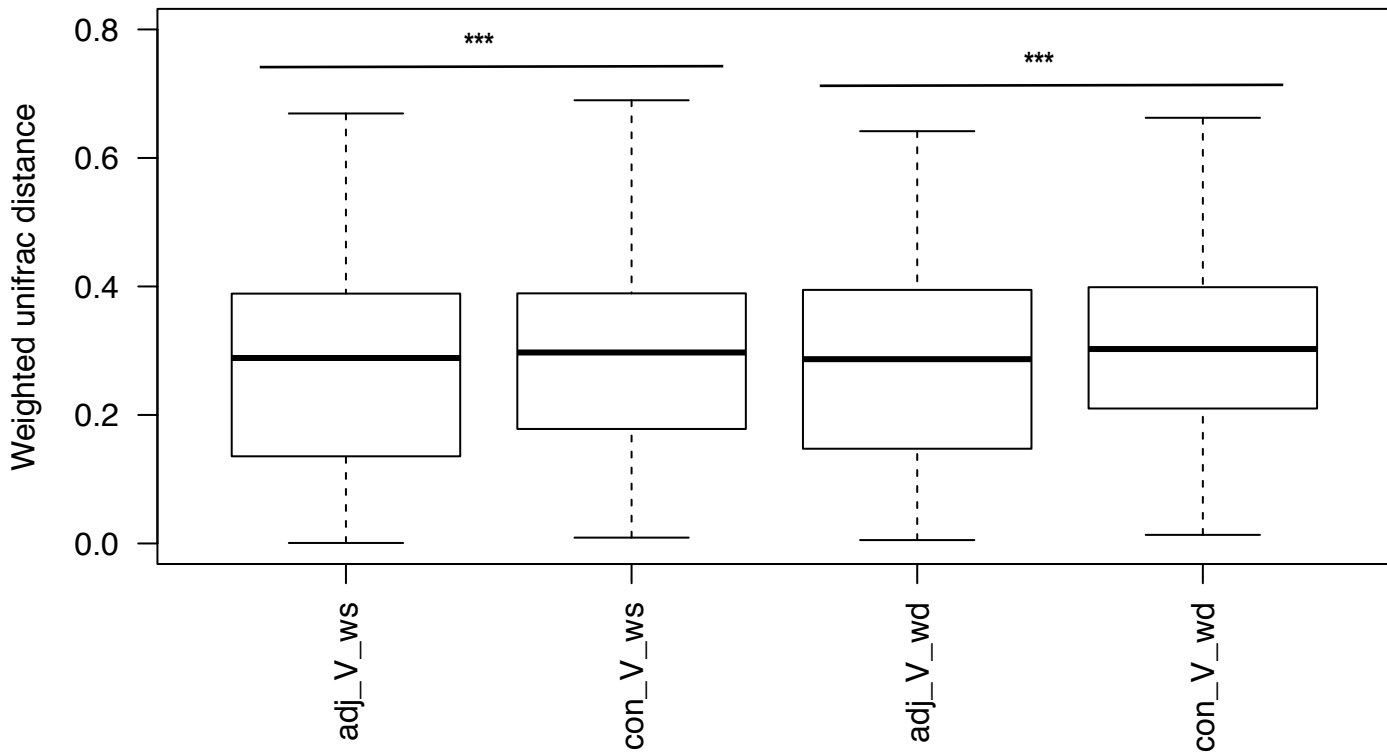

Supplement: Figure S1 — Comparison as adjacent diabetic skin to wound swabs (adj_V_ws), contralateral diabetic skin to wound swabs (con_V_ws), adjacent diabetic skin to wound debridement (adj_V_wd), and contralateral diabetic skin to wound debridement (con_V_ws). Small but statistically significant differences were observed, such that there was greater similarity between adjacent skin and wounds compared to contralateral skin and wounds. Significant differences are indicated with ∗∗∗(p < 0.001). [file peerj-05-3543-s009.pdf]

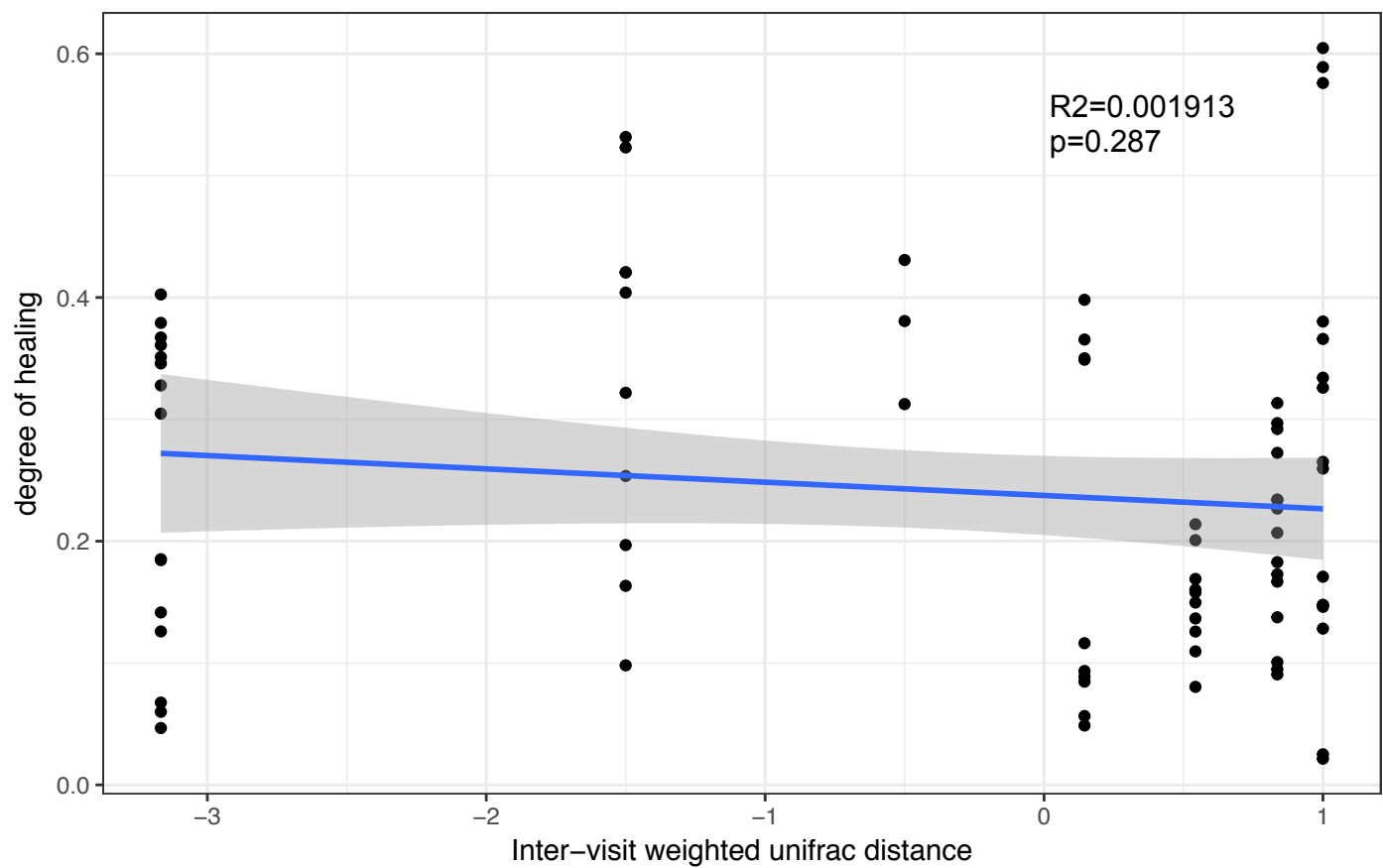

Supplement: Figure S2 — Inter-visit weighted unifrac distances from wound swabs for individual patients were plotted against the degree of healing, where 1 equals complete healing, 0 equals no change in wound size and negative numbers indicate and increase in wound size. No significant correlation was found using a linear model. [file peerj-05-3543-s010.pdf]
